# Bilateral vertical gaze palsy due to midbrain infarct associated with iron deficiency anemia in a young boy

**DOI:** 10.3205/oc000155

**Published:** 2020-06-29

**Authors:** Virna M. Shah, Ratnesh Ranjan, Mrunmayi Jeste, Peter MacIntosh, Duraiswamy Ashwath

**Affiliations:** 1 Aravind Eye Hospital & Postgraduate Institute of Ophthalmology, Coimbatore, India; 2 Ophthalmic Plastic & Reconstructive Surgery and Neuro-Ophthalmology, Illinois Eye and Ear Infirmary, Chicago, USA; 3 Department of Pediatrics, Kovai Medical Centre and Hospital, Coimbatore, India

**Keywords:** vertical gaze palsy, midbrain infarction, iron-deficiency anemia

## Abstract

An 8-year-old boy presented with complaints of sudden-onset binocular vertical diplopia of one day duration. Ophthalmic examination showed restricted up- and downgaze movement with rotatory nystagmus. Systemic investigations revealed iron-deficiency anemia and localized acute infarct in the left paramedian rostral and dorsal part of the upper midbrain at the level of the red nucleus on magnetic resonance imaging. The patient was started on oral iron supplement, which resulted in symptomatic as well as clinical improvement after 2 weeks.

## Introduction

Isolated midbrain infarction, a previously underdiagnosed entity, is being more commonly reported in the era of magnetic resonance imaging (MRI) [1]. Eye movement disorders are the most common signs of midbrain infarction seen in almost two-thirds of cases, with vertical gaze palsy being the most common manifestation [1]. Cardio-embolism and vascular thrombosis are main mechanisms for brain tissue infarction, with hypertension, ischemic heart disease, hyperlipidemia, diabetes mellitus, smoking, and peripheral vascular disease being the common risk factors [1]. Though iron-deficiency anemia (IDA) is not a common cause of stroke, studies have shown a significant association between prior IDA and ischemic stroke [2]. Association of IDA and ischemic stroke has been studied in greater depth among children, and it has been found as an important risk factor for ischemic stroke in otherwise young healthy children [2]. We herein describe a rare case of bilateral vertical gaze palsy due to a unilateral midbrain infarction associated with IDA in a young boy.

## Case description

**Figure 1:**
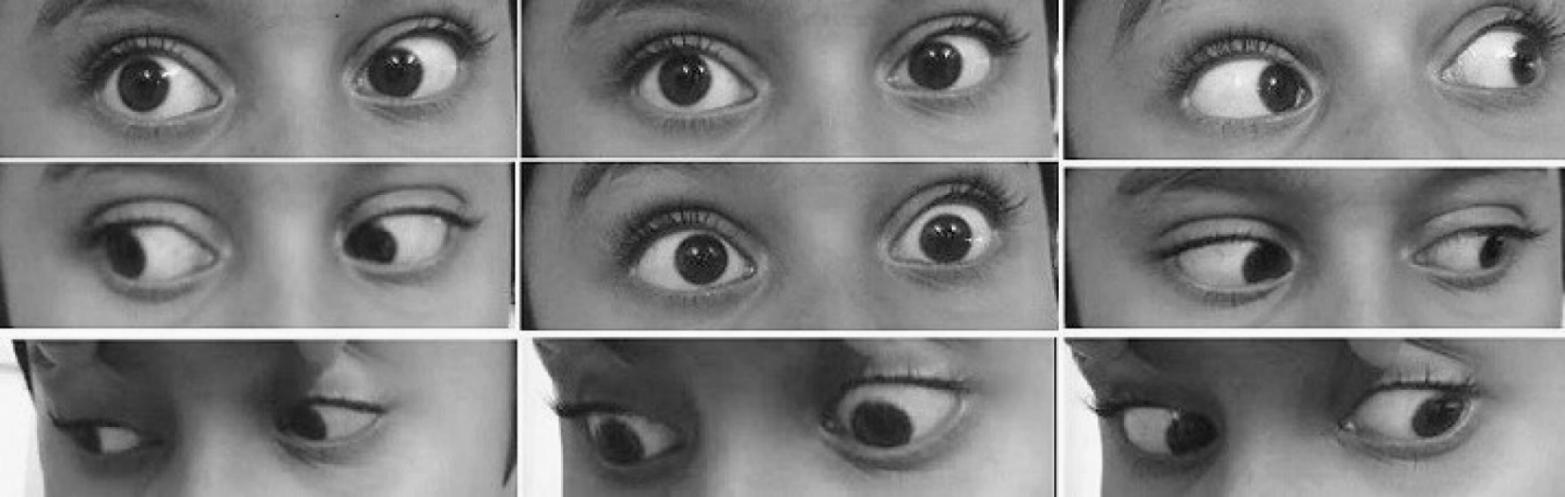
Position of the patient’s eyes in all the gazes at the time of presentation showing significantly restricted ocular movement in upgaze and mild restriction in downgaze

**Figure 2:**
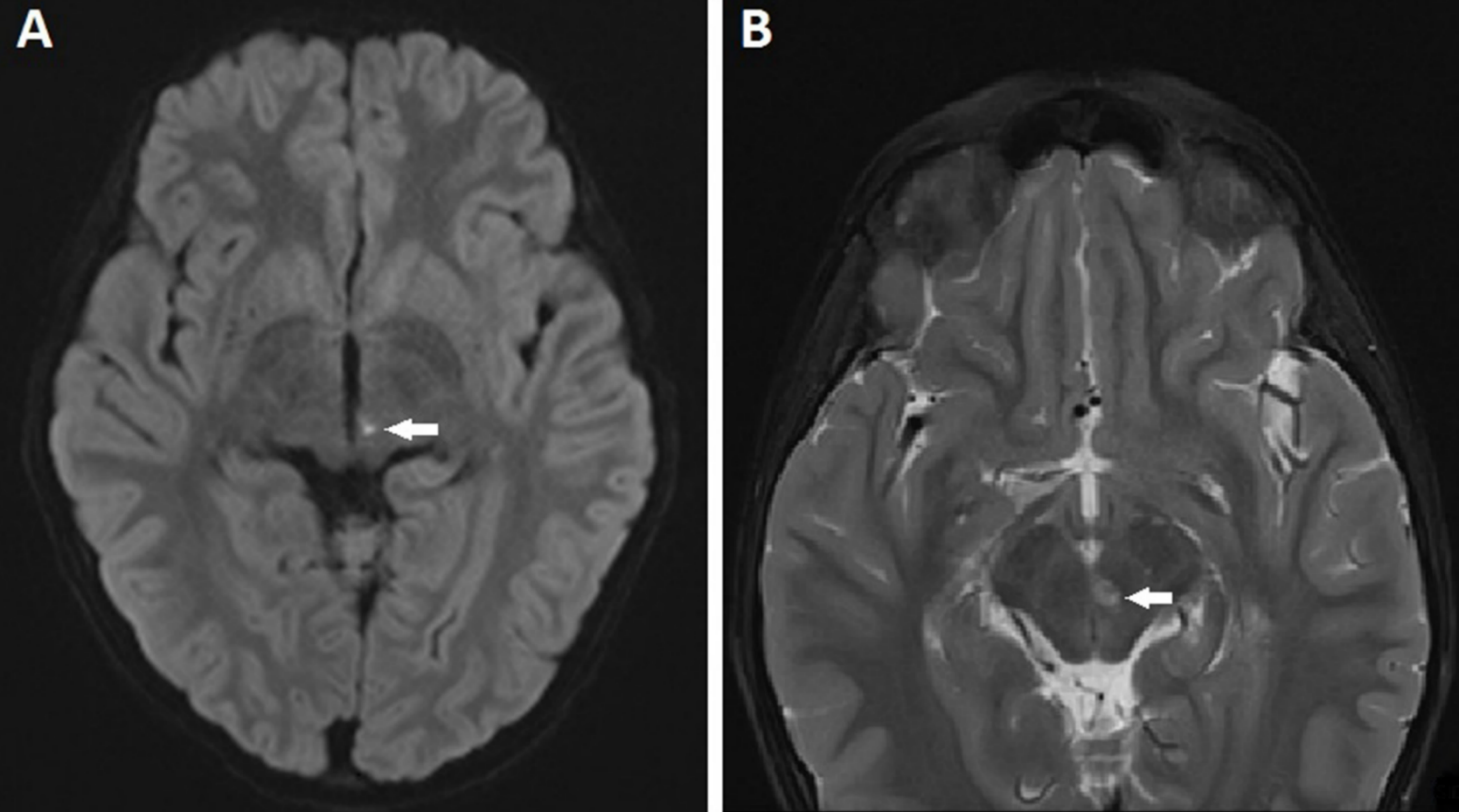
MRI scan of the brain showing tiny infarct (white arrow) in the left paramedian rostral upper midbrain at the level of the red nucleus; (A) axial diffusion weighted image showing restricted diffusion in the T2 hyperintensity, and (B) axial T2 fat saturated image showing T2 hyperintense focus

**Figure 3:**
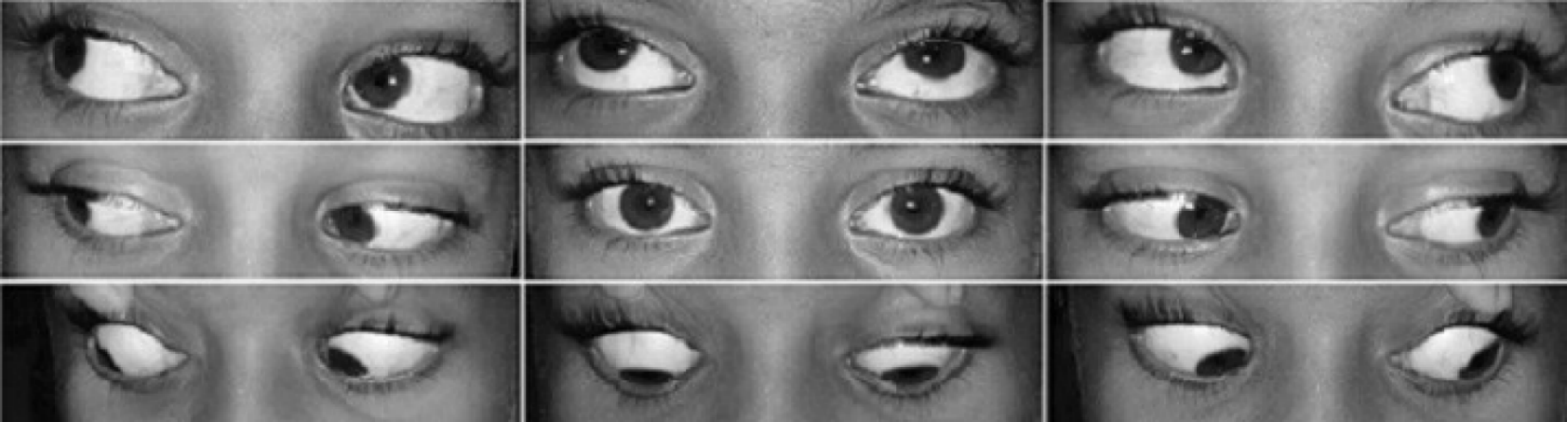
At 2 weeks post-treatment, position of the patient’s eyes in all the gazes showing complete ocular movement

An 8-year-old boy presented to the out-patient department with complaints of sudden-onset binocular vertical diplopia of one day duration. There was no associated significant history except an episode of upper respiratory tract infection one week earlier. General examination demonstrated no abnormalities.The patient’s best-corrected visual acuity was 20/20 in both eyes. Anterior segment examination of both eyes was within normal range, with pupils being round, regular and reactive to light directly and indirectly. Dilated fundus examination was also normal. On motility examination, both eyes showed movement restriction of grade (–4) of upward gaze and (–2) of downward gaze with rotatory nystagmus being present (Figure 1 (Fig. 1)). There were no other neurological abnormalities. Bell’s phenomenon, convergence and horizontal eye movements were completely preserved. An urgent MRI scan of the brain suggested features of localized acute infarct in the left paramedian rostral and dorsal part of the upper midbrain at the level of the red nucleus (Figure 2 (Fig. 2)). Blood investigations revealed hemoglobin 9.9 gm/dl, serum ferritin 6.78 ng/ml, and iron 30 µg/dl with peripheral blood smear showing severe hypochromic microcytic anemia with anisopoikilocytosis being suggestive of IDA. Other blood investigations including random blood sugar, lipid profile, and complete blood cell counts were within normal range. After consultation with a neurologist, a diagnosis of midbrain stroke associated with IDA was made, and the patient was started on cholecalciferol oral drop and sodium feredetate oral syrup. At 2 weeks review, a complete symptomatic as well as clinical improvement was noted (Figure 3 (Fig. 3)).

## Discussion

Vertical gaze palsy resulting from midbrain infarction is usually associated with other neuro-ophthalmologic signs including convergence palsy, nystagmus, ocular tilt reaction due to loss of vestibulo-ocular reflex, and loss of smooth pursuit. The conglomerate of these neuro-ophthalmologic signs depends on the extent of infarct in the paramedian dorso-rostral midbrain at the level of the red nucleus. This part of the midbrain contains closely arranged structures including the rostral interstitial nucleus of the medial longitudinal fasciculus (riMLF), the interstitial nucleus of Cajal (INC), and the posterior commissure (PC), as well as the reticular formation of the midbrain, which play an important role in the mediation of ocular movements, especially vertical gaze. These structures also control the interplay between yoked muscles, interconnection with vestibular control, and light-near reflex [3].

A unilateral lesion of riMLF, INC, and PC can presumptively interrupt the pathways involved in vertical gaze just before decussation of projection from these midbrain structures, resulting in an anatomically unilateral but functionally bilateral lesion [3]. Consequently, there are reports, though not common, describing unilateral midbrain lesion causing bilateral vertical gaze palsy. However, the mechanisms for this phenomenon are not well understood [3], [4], [5]. In our case, the unilateral midbrain infarction only resulted in bilateral vertical gaze palsy with no other neuro-ophthalmologic signs. The small infarct noted on the MRI scan was correlating to the anatomical location of the above-mentioned midbrain structures. However, preserved vestibulo-ocular reflex, smooth pursuit, convergence, and light-near reflex in our case suggests only involvement of riMLF with sparing of INC and PC. A unilateral riMLF lesion might disrupt bilateral upgaze excitatory, inhibitory inputs, and unilateral downgaze excitatory inputs, as suggested by Bogousslavsky et al. [5], based on the autopsy report of their case of conjugate upward and downward gaze palsy showing unilateral infarction of the riMLF, but sparing of the INC and PC. This hypothesis also explains the differential restriction of upgaze and downgaze with the prior being more affected, as seen in our case as well as the case reported by Tsuda et al. [4].

The exact mechanism of IDA-induced stroke is not very clear, although several mechanisms have been explained. A significant decrease in the hemoglobin level in anemic patients possibly compromises the oxygen-carrying ability of the blood flow and subsequently increases the risk of cerebrovascular disease [2]. In addition, increased blood viscosity due to poorly deformable microcytic red-blood cells and secondary thrombocytosis results in a hypercoagulable state, increasing the risk of stroke secondary to IDA. However, an anemic state is well-tolerated to a certain level – unless a triggering condition like viral illness increases the metabolic demands. Relative deficiency of iron-dependent enzymes due to increased metabolic demands results in further impairment of energy metabolism, causing ischemic damage to areas of the brain supplied by end arteries [6]. In our patient, preceding upper respiratory tract infection could be the probable triggering factor for midbrain stroke secondary to pre-existing mild-moderate grade IDA.

In conclusion, our case is unique in presentation with unilateral midbrain infarction, which was of limited extent and severity, manifesting as isolated bilateral vertical gaze palsy with no other neuro-ophthalmic signs. This case highlights the role of detailed investigations for precise recognition of the etiology, and consequent rapid and correct intervention to minimize the morbidity.

## Notes

### Competing interests

The authors declare that they have no competing interests.

### Informed consent

Informed consent for the publication of the patient data has been obtained from the patient’s parents.

## References

[1] Martin PJ, Chang HM, Wityk R, Caplan LR (1998). Midbrain infarction: associations and aetiologies in the New England Medical Center Posterior Circulation Registry. J Neurol Neurosurg Psychiatry.

[2] Chang YL, Hung SH, Ling W, Lin HC, Li HC, Chung SD (2013). Association between ischemic stroke and iron-deficiency anemia: a population-based study. PLoS ONE.

[3] Alemdar M, Kamaci S, Budak F (2006). Unilateral midbrain infarction causing upward and downward gaze palsy. J Neuroophthalmol.

[4] Tsuda H, Maekawa A, Ishihara M (2015). Upward and downward gaze palsy, convergence palsy, concomitant skew deviation and bilateral light-near dissociation due to unilateral rostral and dorsal midbrain infarction. J Med Cases.

[5] Bogousslavsky J, Miklossy J, Regli F, Janzer R (1990). Vertical gaze palsy and selective unilateral infarction of the rostral interstitial nucleus of the medial longitudinal fasciculus (riMLF). J Neurol Neurosurg Psychiatry.

[6] Yager JY, Hartfield DS (2002). Neurologic manifestations of iron deficiency in childhood. Pediatr Neurol.

